# Impact of an integrated control campaign on tsetse populations in Burkina Faso

**DOI:** 10.1186/s13071-017-2609-3

**Published:** 2018-04-27

**Authors:** Lassané Percoma, Adama Sow, Soumaïla Pagabeleguem, Ahmadou H. Dicko, Oumarou Serdebéogo, Mariam Ouédraogo, Jean-Baptiste Rayaissé, Jérémy Bouyer, Adrien M. G. Belem, Issa Sidibé

**Affiliations:** 1 Insectarium de Bobo-Dioulasso – Campagne d’Eradication des Tsé-tsé et Trypanosomoses (IBD-CETT), Bobo-Dioulasso 01, BP 1087 Burkina Faso; 2 Centre International de Recherche-Développement sur l’Elevage en Zone Subhumide (CIRDES), Bobo-Dioulasso 01, 01 BP. 454 Burkina Faso; 3 Ecole Inter-Etats des Sciences et Médecine Vétérinaires (EISMV), Laboratoire d’Endocrinologie et de Radio-Immunologie, BP. 5077 Dakar Fann, Senegal; 4 CIRAD, UMR ASTRE CIRAD-INRA « AnimalS, health, Territories, Risks and Ecosystems », Campus international de Baillarguet, 34398 Montpellier cedex 05, France; 5 Humanitarian Data Exchange (HDX) - humdata.org, OCHA ROWCA regional office. VDN Sacre Coeur III, Villa 9364 BP 16 922, Fann Dakar, Senegal; 6 Laboratoire Régional d’Elevage de Bobo-Dioulasso, Bobo-Dioulasso 01, 01 BP 345 Burkina Faso; 7 CIRAD, UMR INTERTRYP, F-34398 Montpellier, France; 8 Université Polytechnique de Bobo-Dioulasso, Bobo-Dioulasso, Burkina Faso

**Keywords:** Tsetse fly, *Glossina*, Trypanosomosis, Eliminate, Target, Control, Burkina Faso

## Abstract

**Background:**

Tsetse flies are the sole vectors of human and animal trypanosomosis. In Burkina Faso, a project aiming to create zones free of tsetse flies and trypanosomosis was executed from June 2006 to December 2013. After the determination of tsetse distribution in the intervention area from December 2007 to November 2008, the control campaign was launched in November 2009 and ended in December 2013. The goal was to eliminate tsetse flies from 40,000 km^2^ of area, through an integrated control campaign including insecticide targets, traps and cattle, sequential aerial treatment (SAT) and the mass treatment of livestock using trypanocides. The campaign involved assistance of the beneficiary communities at all the steps of the control strategy with insecticide impregnated targets.

**Methods:**

This study was carried out to assess the impact of the control project on tsetse apparent density per trap per day (ADT). To evaluate the effectiveness of tsetse control, 201 sites were selected based on the baseline survey results carried out from December 2007 to November 2008. These sites were monitored bi-monthly from January 2010 to November 2012. At the end-of-study in 2013 a generalized entomological survey was carried out in 401 infested sites found during the longitudinal survey done before the control. Barrier and tsetse persistence areas were treated by ground spraying and evaluated. Controls were also done before and after aerial spraying.

**Results:**

In the insecticide-impregnated target area, the control showed that ADT of tsetse flies declined from 10.73 (SD 13.27) to 0.43 (SD 2.51) fly/trap/day from the third month of campaign onwards (*P* < 0.0001) and remained low thereafter. At the end of the campaign in 2013, an 83% reduction of ADT was observed for *Glossina palpalis gambiensis* and a 92% reduction for *G. tachinoides*. Tsetse flies were captured only in 29% of the sites found infested in 2008.

**Conclusions:**

Tsetse flies could be suppressed efficiently but their elimination from the targeted area may require the use integrated methods including the Sterile Insect Technique, which is programmed through the development of the Pan African Tsetse and Trypanosomiasis Eradication Campaign (PATTEC Burkina) insectarium. The challenge will remain the sustainability of the achievement.

**Electronic supplementary material:**

The online version of this article (10.1186/s13071-017-2609-3) contains supplementary material, which is available to authorized users.

## Background

The national component of the Pan African Tsetse and Trypanosomosis Eradication Campaign (PATTEC) is the most important tsetse control program ever implemented in Burkina Faso. Its intervention area covers 96,600 km^2^ for control and 40,000 km^2^ for total eradication. The current program took advantage of lessons learned from the past and was based on a holistic approach of trypanosomosis control. Indeed, the main reason for failure of past campaigns in Burkina Faso was the sustainability of the achievement. In the 1980s and 1990s, numerous tsetse control projects were implemented, namely in the agro-pastoral zone of Sidéradougou [1] and the pastoral zone of Yalé [2, 3]. In these campaigns, beneficiary communities, farmers and public Authorities did not continue the efforts after the projects ended. Consequently, the tsetse cleared areas were re-invaded rapidly by tsetse and the trypanosomosis incidence regained similar levels as before the projects implementation. The successes and failures of these projects have been discussed previously [4].

For tsetse control, an integrated pest management, including impregnated targets and traps, live baits (insecticide treated cattle), ground spraying and sequential aerial treatment (SAT), was used by PATTEC Burkina. Barriers were used between treated and untreated areas. The campaign funded the mass treatment of livestock against trypanosomosis. One of the most important components of this campaign was the involvement of beneficiary communities.

This study aimed to describe the various tsetse fly control actions carried out and to assess their effectiveness to control tsetse and trypanosomosis.

## Methods

### Study area

**Fig. 1:**
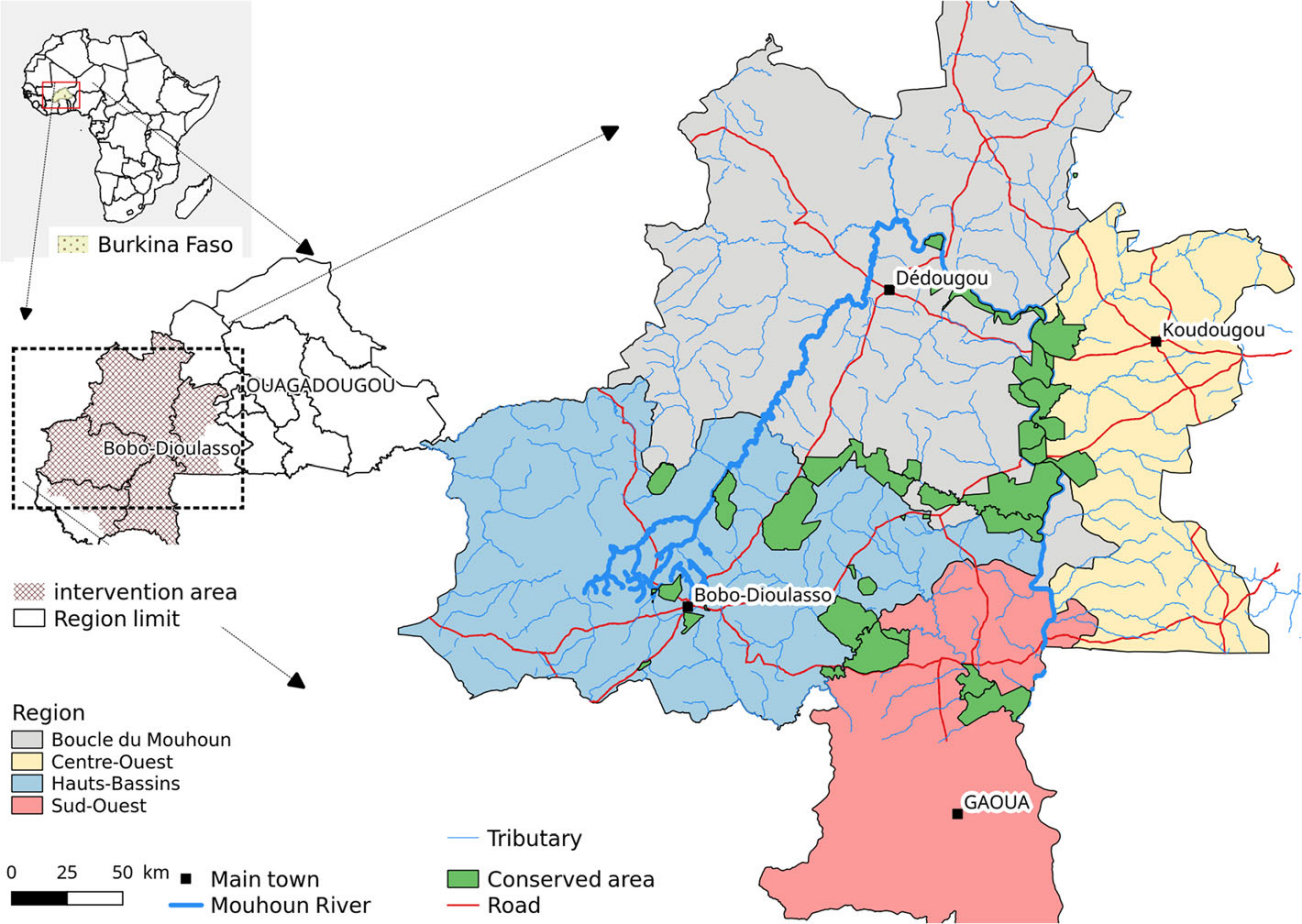
PATTEC intervention area in Burkina Faso

The study was carried out in the regions of Boucle du Mouhoun, Hauts Bassins, Centre-Ouest and Sud-Ouest (aerial spraying area), covering the PATTEC intervention area of Burkina Faso (Fig. 1). These regions are mainly rural, where most of the population earns their life by crop production and livestock husbandry [5]. They host almost 700,000 cattle, double this number of small ruminants, and tens of thousands of pigs, horses and donkeys [6]. The region is one of the areas in the country where trypanosomosis is the most prevalent [7]. The Mouhoun River crosses the region over almost 280 km, describing a loop. The main river is joined by permanent secondary tributaries. This explains the abundance of riverine tsetse species, *Glossina palpalis gambiensis* and *G. tachinoides* [8]. The entomological surveys funded by PATTEC Burkina and conducted before the control measures showed that the apparent abundance per trap was high, especially on the main river and permanent tributaries, where more than 92 tsetse/trap/day were observed in some sites [9].

### Trypanosomosis and tsetse control strategies

#### Communities’ awareness and their participation

Tsetse control involved communities’ commitment [4, 10]. For the current PATTEC Burkina campaign, the communities were sensitized on trypanosomosis socio-economic impact and the different control methods. During the entomological baseline surveys, they were used as guides. After the entomological baseline survey and during the control, populations of 300 infested villages, grouped in 53 village clusters, were regularly informed about the results obtained and awareness was stimulated. In each village, at least 5 people were chosen as auxiliaries for PATTEC Burkina field teams in order to assist them in the deployment and surveillance of targets impregnated with insecticide. They carried out an important part of the field work, including the setting of insecticide impregnated targets during the dry season and their withdrawal or moving during the rainy season. Additionally, four supervisor teams of barriers were created in conjunction with the municipal authorities of these barriers and equipped with some working materials including bicycles, boots, wires, cutters, etc.

#### Insecticide impregnated targets

Targets used for the control campaign were manufactured by the Vestergaard Frandsen Company (Lausanne, Switzerland). They were made with polyester material. Targets consisted of a central blue rectangular piece (50 × 100 cm) flanked by two black strips (25 × 100 cm); hence, the total surface of a target was 1 m^2^. According to the manufacturer, those deltamethrin impregnated targets are effective against tsetse for 2 years. All rivers and tributaries found infested during the baseline entomological survey were covered by impregnated targets and each target deployed was geo-referenced according to the Universal Transverse Mercator coordinate system (UTM). Targets deployed remained on the field all year.

**Fig. 2:**
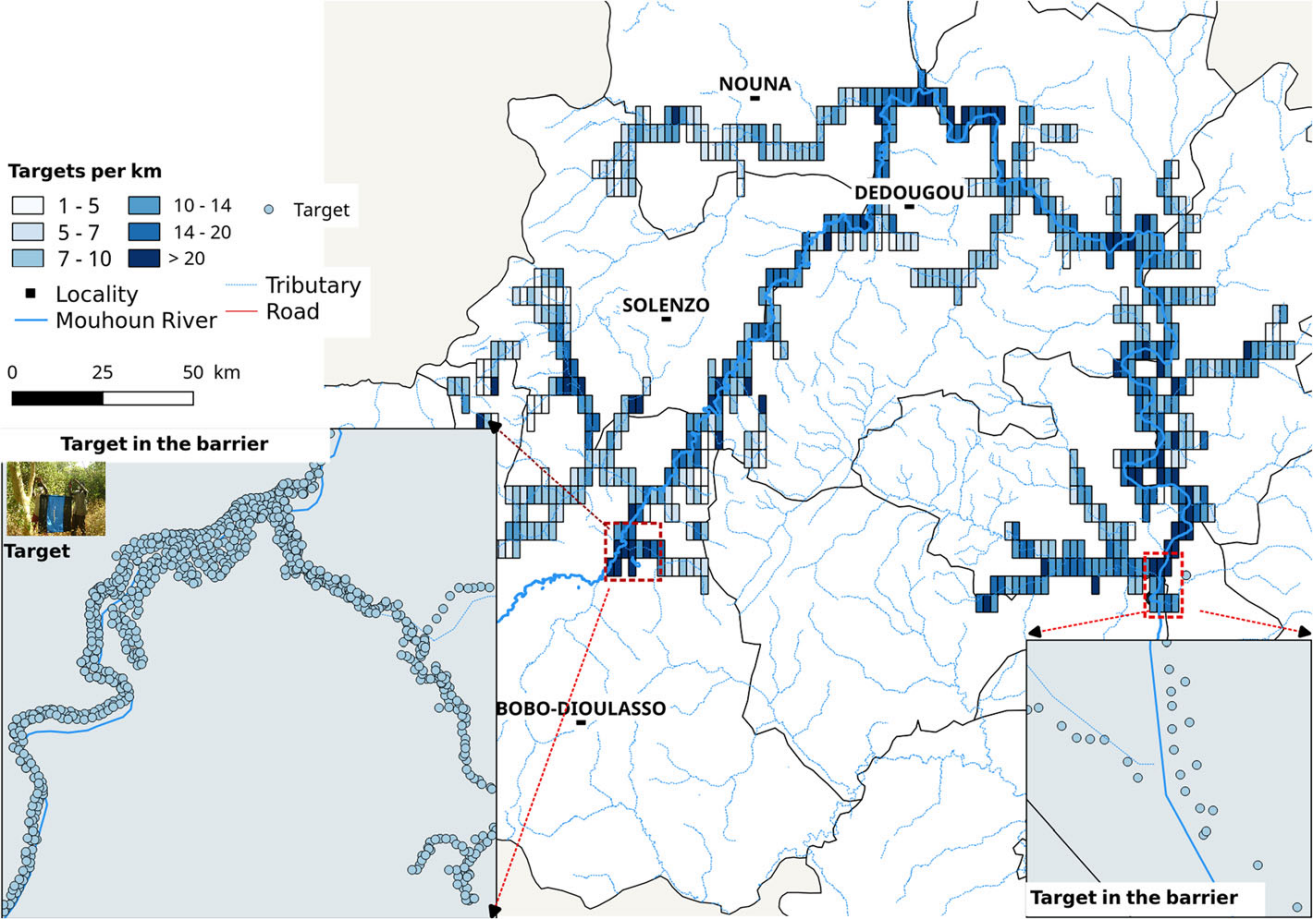
Insecticide targets density along the Mouhoun River and its main tributaries

From November 2009 to April 2010, 21,360 impregnated targets were deployed (Fig. 2). The targets were set alternatively on one of the two banks of the main river and some main tributaries, and on one bank of the small tributaries, at distances of 50–100 m between targets. Sometimes selective bush clearing was necessary to increase the target visibility. Targets were fixed either on metallic poles or hung on tree branches over the river.

To calculate target density per km, 5 × 2 km grids were realized along covered rivers. The density per km of river was obtained by the formula: $\frac{Number\ of\ targets\ of\ each\ grid}{length\ of\ river\in each\ grid}$ and km^2^ of surface by $\frac{Number\ of\ target\in each\ grid}{area\ of\ a\  grid\kern.2em \left(k{m}^2\right)}$ by spatial analyses of the data extracted from a relational database.

In 2011 and 2012, 15,562 and 3726 targets were deployed respectively to replace the ones which disappeared or to reinforce them. In total, from November 2009 to 2012, 42,138 targets were deployed.

#### Barriers

Previous genetic studies showed that the target area was not isolated [11–13]. Following the recommendations of Kgori et al. [14], the target area was thus protected from re-invasion using artificial barriers. Two barriers were built from May, 31th to June, 04th 2010 on the two branches of the Mouhoun River (ascendant and descendant) by impregnated targets deployment (Fig. 2). On the ascendant branch, the “Satiri” barrier was built with 483 impregnated targets and 78 impregnated traps (biconical and monoconical) on a distance of 8.3 km along the main river and 293 impregnated targets and 39 impregnated traps on 6.8 km along the tributaries. On the descendant branch, the “Fara” barrier was made with 95 targets on 9.64 km of Mouhoun River and 74 targets on 5.53 km of tributaries. This barrier had benefited of sequential aerial treatment done between Ghana and Burkina Faso. The local supervisors maintained regularly the barriers. Destroyed targets were replaced twice a year at the beginning (June) and end of the rainy season (November-December) by PATTEC technicians.

#### Sequential aerial treatment

**Fig. 3:**
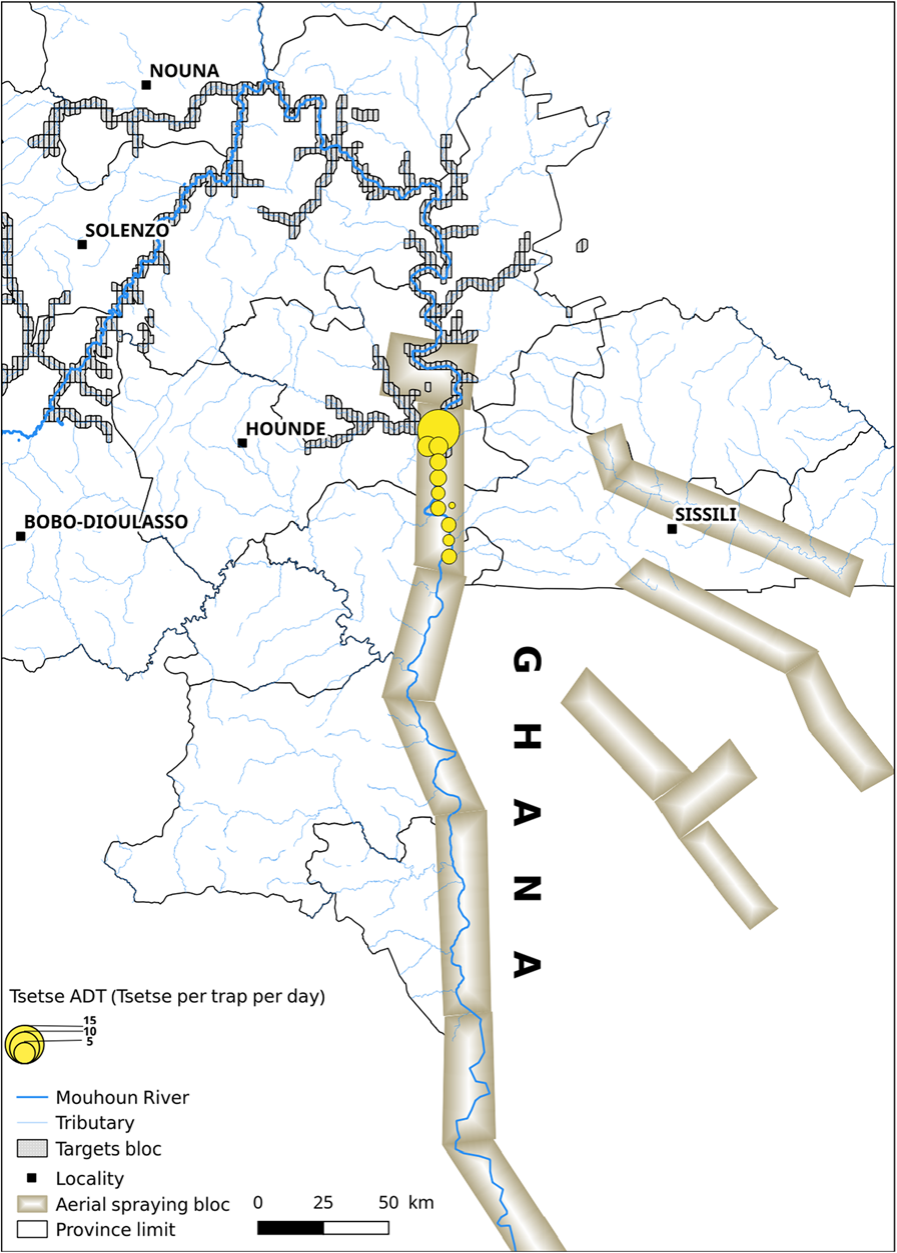
Location of the sequential aerial spraying between Ghana and Burkina Faso

Sequential aerial treatment was carried out jointly with the counterpart of Ghana [15]. The main objective was to limit or to avoid the re-invasion between the two countries intervention areas. It concerned the descendant part of the Mouhoun River (Fig. 3) on 114 km and 138 km on Sissili River. Seven km were sprayed on both sides of every bank. Aerial spraying and targets covered a same area on around 79 km of river. The campaign used special equipped airplanes hired from ORSMOND Aviation, South Africa [15]. In Burkina Faso, the SAT operations were conducted between 26th of March and 9th of May 2010 at the beginning of the rainy season. The applied insecticide was Deltamethrin (0.35% (*w*/*v*), ultralow volume (ULV) (Deltanex formulation, Avima, Johannesburg, South Africa). Application rate was 0.33–0.35 g active ingredient (a.i)/ha at about 10 m above tree canopy [15]. The dispersal units were operated with cage speed of 11,000 rpm and an average flow rate of 9.7 l/km^2^ [15]. Temperature inversion was investigated at the airfield, but not in the gallery forest, using probes located at a height of approximately 1.5 and 8 m. This inversion layer was shallow in the early afternoon, improving through the night and breaking down around 07:00 am [15]. Four weekly sequential aerial treatments were carried out. For the monitoring, entomological data were collected before and after spraying operations with a variable number of Challier-Laveissière traps. The raw data are presented in Additional file 1: Table S1.

#### Ground spraying

Ground spraying was used in the Satiri barrier area before every rainy season to reinforce it, in bushy areas and tsetse persistence zones at the end of project (Table 1). Some areas inaccessible from the ground were sprayed using a boat. Indeed, after the first phase of the generalized entomological survey (from 22nd January to 05th February 2013), tsetse seemed to persist on the “Siou River” and some of its tributaries in the western part of the intervention area (towards the border with Mali). A total of 4.08 km^2^ of gallery forest were sprayed three times from 10th to 24th March, 07th to 21st April and 05th to 12th May. Seven sites were chosen for monitoring with biconical traps before and after spraying. Insecticide was applied on ultra-low volume using Swingfog® foggers (Isny, Germany; SN 50 and SN 101), or PortaPak sprayers and by nebulization with Mist Blowers at 3 g/ha. The insecticide used was deltamethrin, (Aqua-k-othrin®, Leverkusen, Germany; 2% or K-Othrine 2.5%). Fogging was applied early in the morning and late in the evening (after 4 pm).

**Table 1:** Surface of treated zones by ground spraying

Treated zone		Length of river (km)		Swath width (m)	Total length treated (km)	Surface treated (ha)
		Right bank	Left bank			
Barrier	Mare aux hippopotames	2	2	50	4	20
	Zangoma	1	5	50	6	30
Tsetse fly persistance zones	Lery-Kouri	10	10	50	20	10
	Dedougou	8	8	50	16	80
	Darsalam	4	4	50	8	40
	Tansila	7.5	7.5	50	15	75
	Lery-Bouni	14	14	50	28	140
	Siou river and its tributaries	40.63	40.43	50	81.06	405.3

#### Trypanocide and epicutaneous mass treatment

**Fig. 4:**
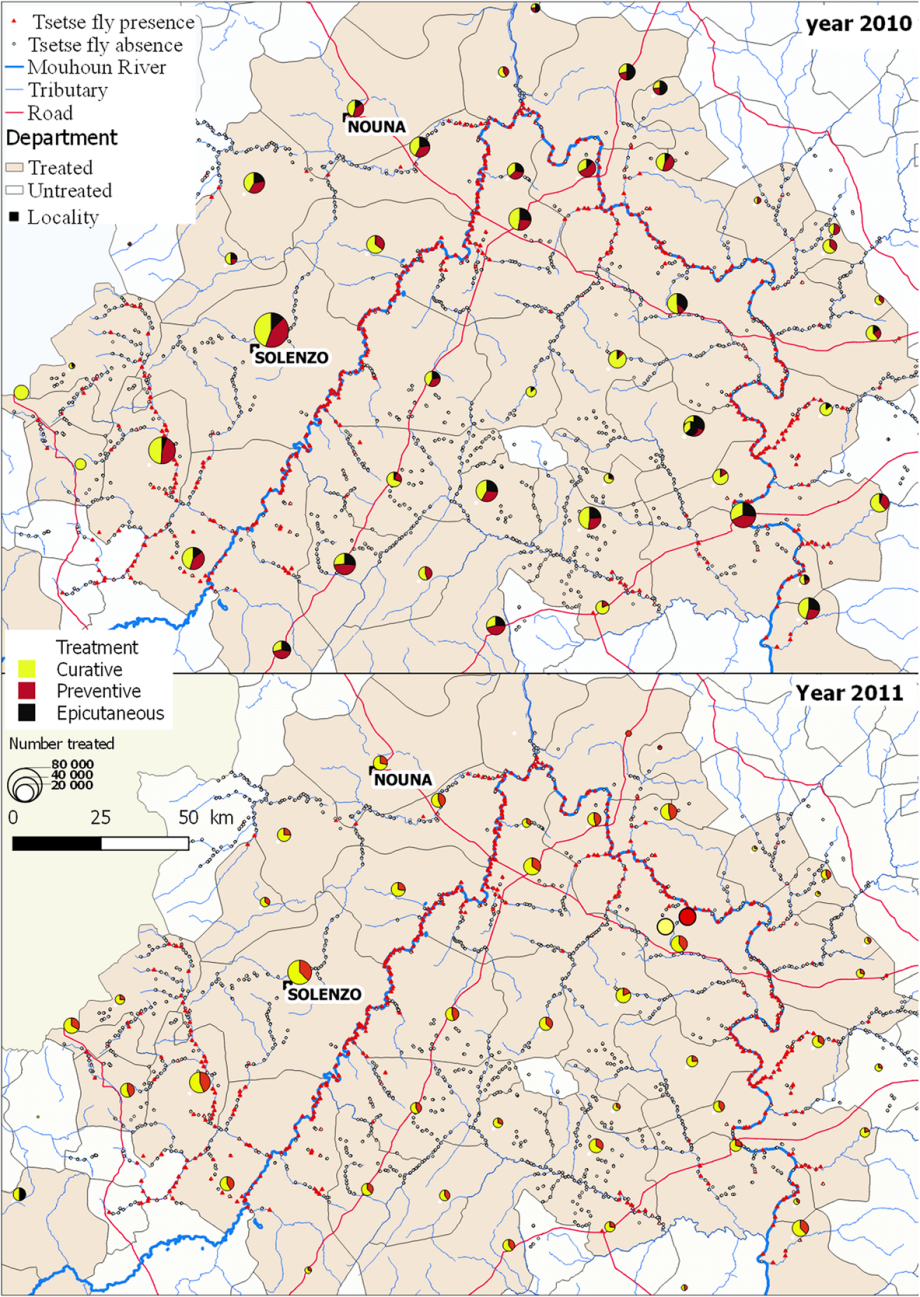
Distribution of trypanocide and mass epicutaneous treatment of cattle in the study area

Mass epicutaneous (pour on and spray) and trypanocide treatment of cattle, sheep and donkeys was carried out by private veterinarians (Fig. 4). The main objective was to contribute to avoid trypanosomes transmission. The trypanocide treatment consisted to curative treatment with diminazene aceturate (3.5 mg/kg b.w. deep intramuscular injection) followed by a preventive treatment three weeks period after with isometamidium (1 mg/kg in 2% solution). Stockbreeders were required to apply epicutaneous treatments with cypermethrin 15 g/l. Treated animals constitute live baits for tsetse. It concerned the animals of villages located within a zone of 10 km on both sides of infested rivers. In 2010, all treatments were supported by PATTEC. The participation of the communities was on a voluntary basis. In 2011, only the cost of the trypanocides was financially supported by PATTEC; veterinarian’s services had to be paid by farmers. The treatments were carried out during the rainy season (from June to December). More than one million animals were treated (Table 2).

**Table 2:** Trypanocide and mass epicutaneous treatments in the control area

Year	No. of departments	No. of producers	Number of animals (*n*)			
			Curative treatments	Preventive treatments	Epicutaneous treatment	Total
2010	60	19,728	512,668	375,296	238,738	1,126,702
2011	59	10,799	380,088	208,799	60,064	648,951

### Periodical entomological survey

**Fig. 5:**
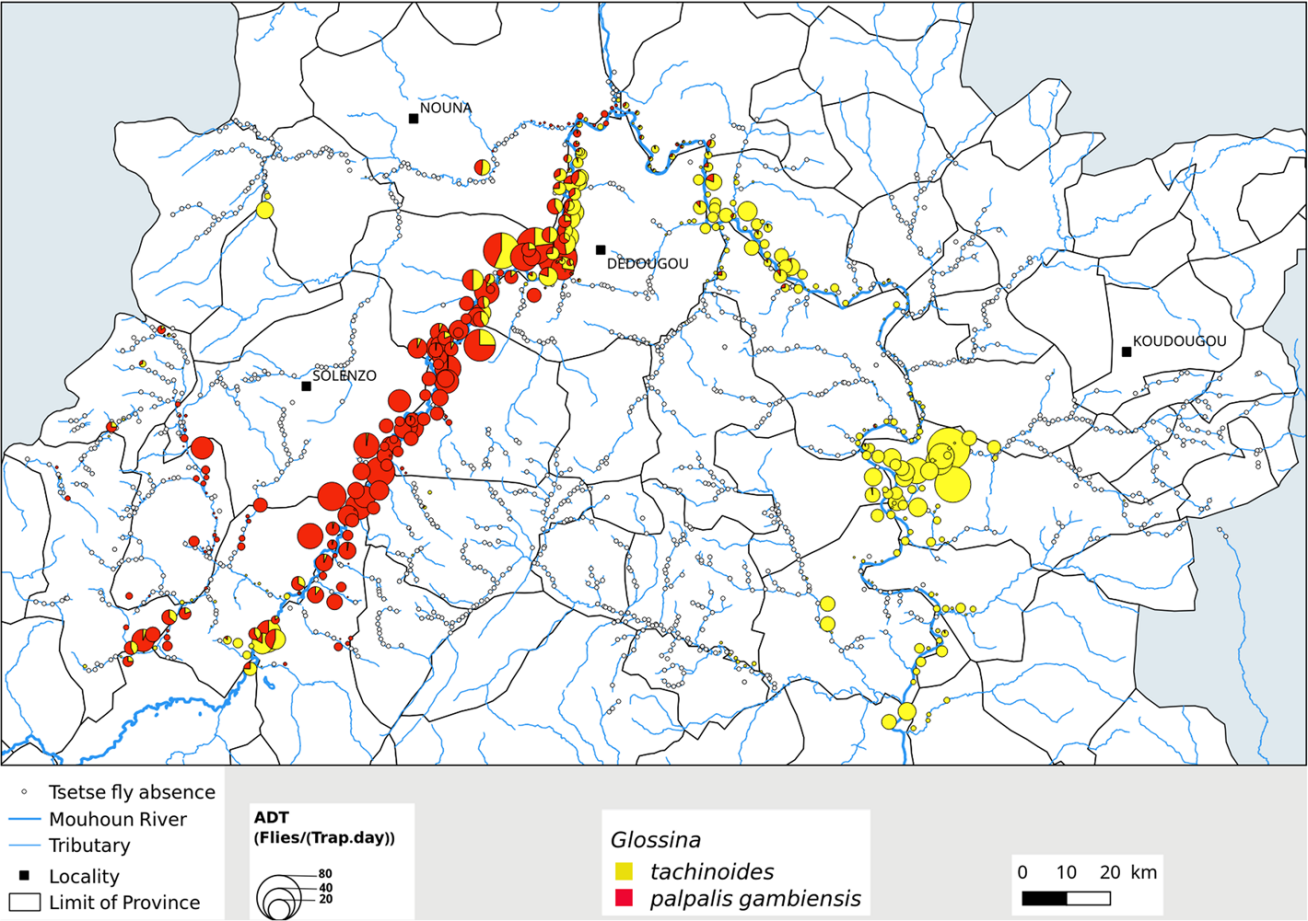
Location of sentinel traps

More than 201 monitoring sites were selected based on the entomological baseline survey carried out from December 2007 to November 2008 [9]. Selection criteria were the abundance of tsetse flies (apparent densities per trap per day (ADT)) and the spatial relationship between sites (a maximum of 4 sites per square cell of 10 × 10 km) (Fig. 5). Every two months, two sentinel traps were set in each monitoring site. All trapping sites were geo-referenced. To increase the attractiveness of the traps, a dispenser containing a 1:4:8 mixture of 3-n-propylphenol, 1-octen-3-ol and para-cresol was attached at the bottom of the trap. The pockets used to release the chemicals were made of 0.15 mm thick polyethylene measuring 4 × 4 cm, providing a diffusion surface of 32 cm^2^ [16]. Each pocket contained 2 ml of mixture. In 2004, in an experience on the Sissili and Mouhoun River, this mixture increased the trapping of *Glossina palpalis gambiensis* and *G. tachinoides*, both Palpalis group flies, by a factor of two for both sexes compared to the trap alone [16]. Traps were set for 3 days before collection. Trapped insects were counted, identified by species and sex, and recorded in a data sheet. The raw data are presented in Additional file 2: Table S2.

### Longitudinal parasitological survey

This study concerned sedentary cattle only. Based on the cross-sectional study in the PATTEC intervention area [17], 11 villages where the disease was more prevalent were selected for the longitudinal survey. However, two villages were abandoned later because of the absence of animals during the sampling periods and replaced by new ones. In each village, a sentinel herd of 50 cattle was selected. All sentinel animals were ear tagged. At the beginning of the survey, sampled cattle were treated with diminazene aceturate (DA), and dewormed with albendazole (Vermitan®, CEVA, Libourne, France) (7.5 mg/kg). They received every two months a pour-on formulation mixing cypermethrin and amitraz (Cypertraz®, CEVA, Libourne, France) [18].

Before receiving any veterinary care or treatment, blood samples were collected from each animal through a jugular vein puncture with an EDTA tube for the detection of motile trypanosomes using the buffy coat technique [19]. Bimonthly monitoring surveys were carried out in all villages, in the view to check trypanosomosis infections in sentinel herds. During each survey, ill animals were treated with DA at the dose of 3.5 mg/kg b.w. Animals which missed one bimonthly follow-up were removed from the study onward. Sampling was also carried out on some calves in additional to the 50 heads of adult animals. To avoid self-medication by farmers, it was required from them to contact a PATTEC field veterinary technician who was made available throughout the study for any health care for the sentinel cattle.

### Final generalized (transversal) entomological survey

From the beginning of tsetse control to December 2012, longitudinal entomological survey were conducted. At the end, in May 2013, a generalized control (transversal) was conducted in all areas. 401 sites found infested by tsetse before control were concerned.

### Data analysis

Data were stored in a relational database to facilitate analysis. As described previously [20], we used a generalized linear mixed model [21] to measure the impact of the suppression on tsetse apparent densities (ADT) and trypanosomosis prevalence. For ADT, the response data were tsetse apparent densities/trap/day. Time (measured in years), species (*G. p. gambiensis* and *G. tachinoides*), the river section (RAB, right ascendant branch; LAB, left ascendant branch; LAB; RDB, right descendant branch; and LDB, left descendant branch), and their interactions were used as fixed effects, whereas the trap locations were used as random effects. Raw data for ADT are presented in Additional file 2: Table S2. For the trypanosomosis prevalence, time, locality and animal status (adult or calf) were used as fixed effects, whereas the animal numbers were used as random effect. Raw data for trypanosomosis prevalence are presented in Additional file 3: Table S3. For both analyses, the best model was selected on the basis of the lowest corrected Akaike information criterion (AICc), and the significance of fixed effects was tested using the likelihood ratio test [22, 23].

Moreover, the tsetse apparent densities (ADT) before and after the control campaign in the all area were compared using a paired Wilcoxon rank sum test for both species [24]. Raw data of these two surveys are presented in Additional file 4: Table S4.

## Results

### Impregnated target distribution

The maximum density of targets was 42 km^2^ with an average density of 3.12 (SD 3.32). The lowest densities were recorded in the lateral grid where tsetse infestations were weak and the highest on Mouhoun River different branches, especially at the top and the barriers. The density per km of river was 13.82 (SD 29.09).

### Longitudinal monitoring of tsetse densities

**Fig. 6:**
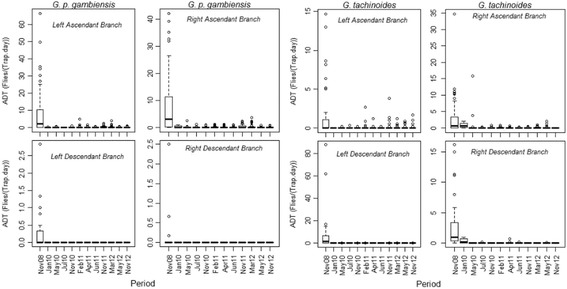
Dynamics of tsetse apparent densities in the target area by river section. Tsetse densities during the fighting against tsetse fly in impregnated target area in different periods, the data are presented by species and river section. Boxplots present the median (bold line), quartiles (boxes), 95% confidence intervals (horizontal lines) and erratic values (circles)

The dynamics of tsetse apparent densities (ADT) in the monitoring system are shown in Fig. 5. Only two months after the setting of impregnated targets, the tsetse ADT dropped by more than 90%. The rate of reduction reached 95% within only 3 months of control. A high level of reduction of 99.77% was obtained in June 2011 for *G. tachinoides* and 99.84% in July 2010 for *G. palpalis gambiensis*. A new increase of the density of *G. tachinoides* was noted in the Left Ascendant Branch (LAB) from February 2011 to December 2012 (Fig. 6).

This reduction of the ADT with time was highly significant for both species (Table 3; *P* < 0.0001). The apparent density of *G. palpalis gambiensis* was overall higher than that of *G. tachinoides* at the beginning of the control campaign (*P* = 0.002) and dropped faster for this species (*P* = 0.004). Global tsetse densities were lower on the descendant branch and the tributaries located on its right bank (RDB) than in the other river sections at the beginning of the control campaign (Table 3).

**Table 3:** Fixed-effect coefficients for the AICc-best Linear mixed-effects model of the dynamics of tsetse apparent densities

Parameters	Value	SE	*t*-value^a^	*P*-value
(Intercept)	2.554	0.200	12.821	< 0.0001
Time	-0.602	0.053	-11.445	< 0.0001
Species *Gpg*	0.791	0.260	3.037	0.002
Section LAB	0.020	0.132	0.148	0.882
Section RDB	-0.476	0.136	-3.498	0.001
Section LDB	-0.216	0.126	-1.710	0.087
Time: species *Gpg*	-0.212	0.074	-2.846	0.004

^a^Degrees of freedom = 3895

*Glossina tachinoides* were used as references for analysis

*Abbreviations*: *Gpg Glossina palpalis gambiensis, LAB* left ascendant branch, *RDB* right descendant branch, *LDB* left descendant branch, *RAB* right ascendant branch, *SE* standard error

### Final generalized (transversal) entomological survey

**Fig. 7:**
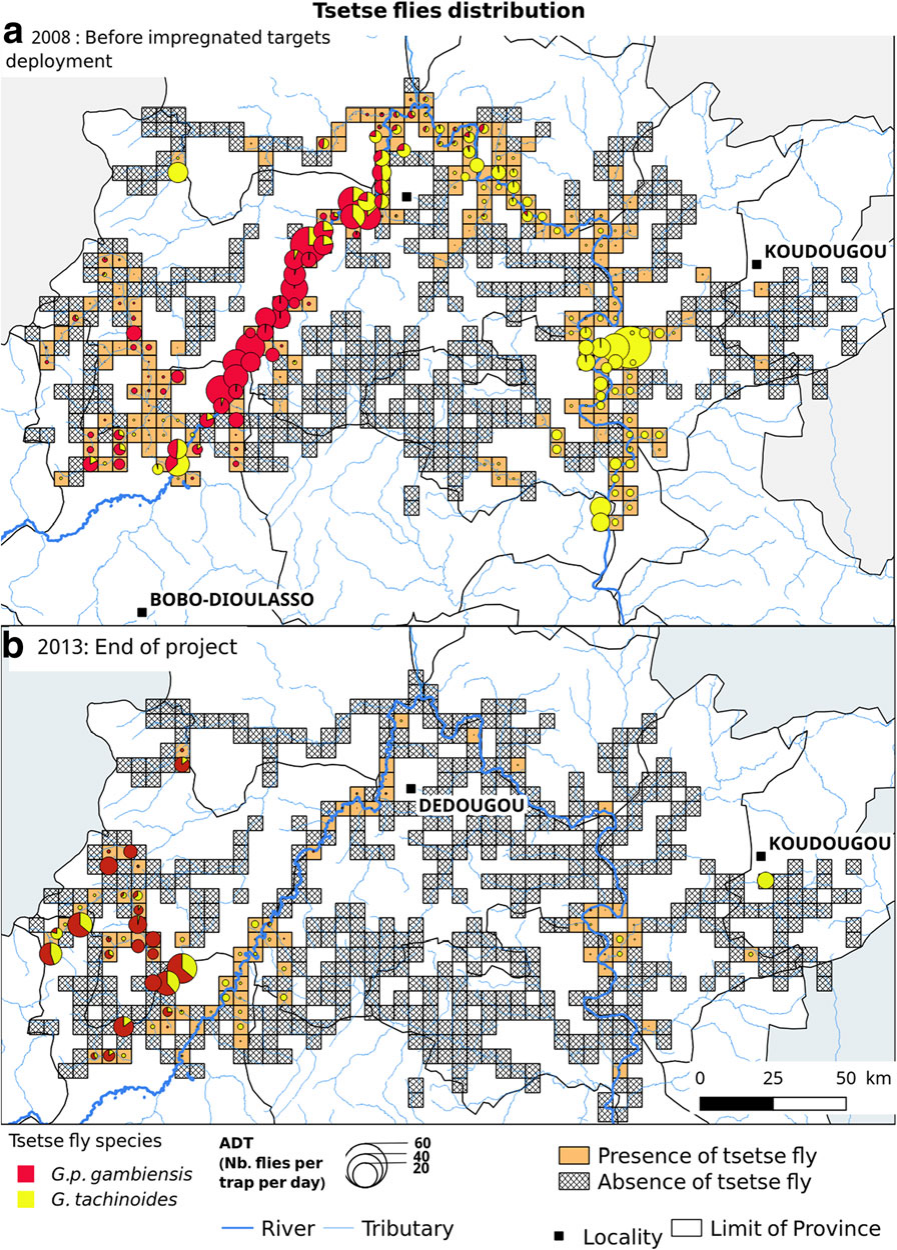
Tsetse distribution before (**a**) and at the end of the project (**b**)

When comparing the densities before (October 2008) and after (May 2013) the control campaign in all those sites, where tsetse flies were present before the start of control operations, a strong reduction of ADT was observed (Fig. 6; *P* < 0.0001). For *G. palpalis gambiensis*, ADT dropped from 3.337 (SD 7.998) to 0.565 (SD 2.795) (83% reduction, Table 4, *P* < 0.0001). For *G. tachinoides,* they dropped from 2.888 (SD 7.119), to 0.230 (SD 1.256) (92% reduction, *P* < 0.0001). In 2013, tsetse flies were captured only in 29% of the sites found infested in 2008. However, some high infestations were found in the Western part of the intervention area (Fig. 7).

**Table 4:** Results from comparisons of tseste ADT before and at the end of the project (one-tailed Wilcoxon signed rank test with continuity correction)

V-statistic (sum of positive ranks)	*P*-value	Test type
8701	< 0.0001	Global
3176	< 0.0001	*G. palpalis gambiensis*
8220	< 0.0001	*G. tachinoides*

### Impact of ground spraying on tsetse apparent density on the Siou River

**Fig. 8:**
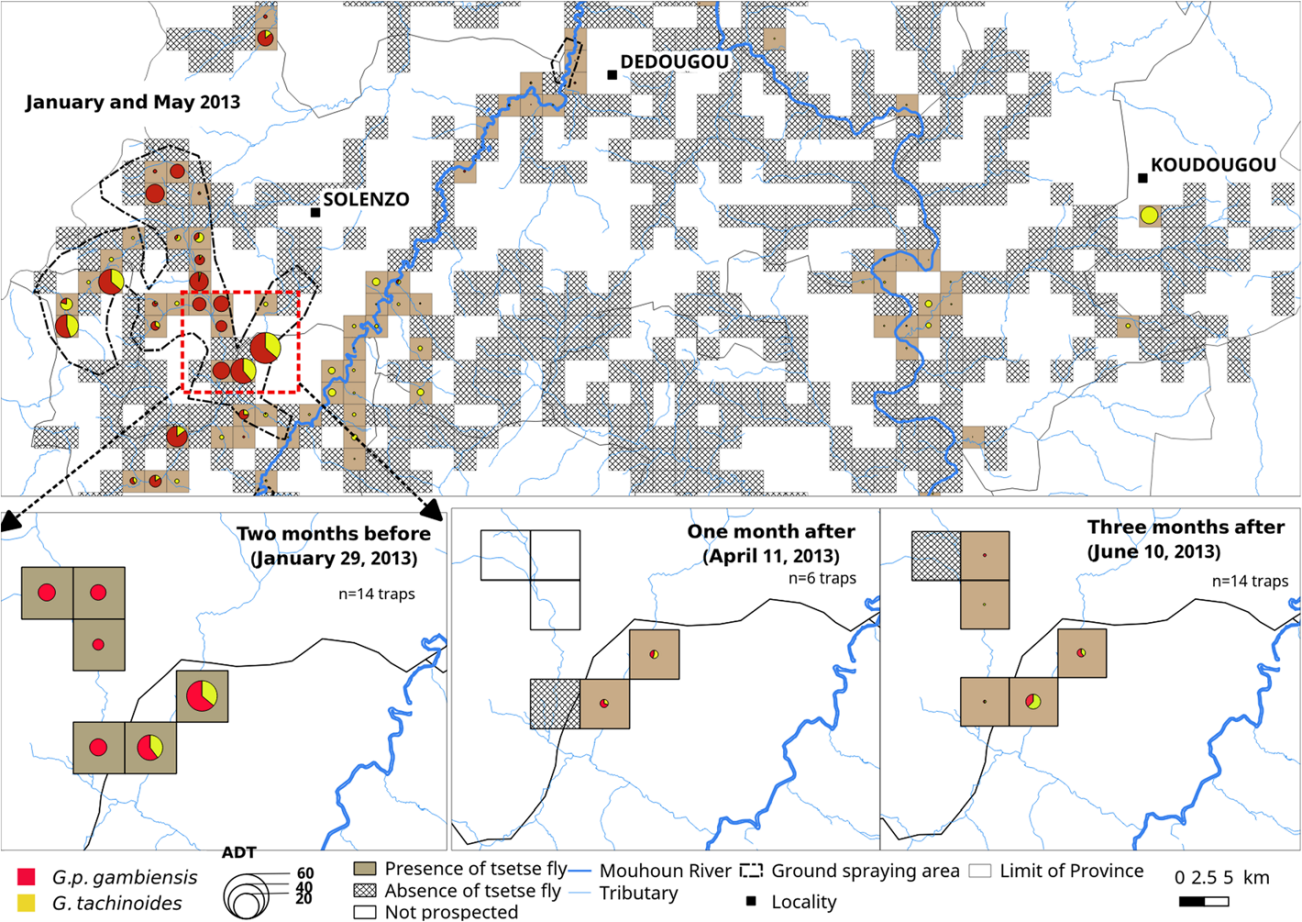
Dynamics of tsetse flies apparent densities per trap per day (ADT) before and after ground spraying

Some rates of reduction of 82.82 and 61.96% were noted one and three months after spraying for *G. tachinoides*, respectively, and 92 and 90% for *G. palpalis gambiensis*. Figure 8 shows the dynamics of densities.

### Impact of aerial spraying on tsetse ADT

**Fig. 9:**
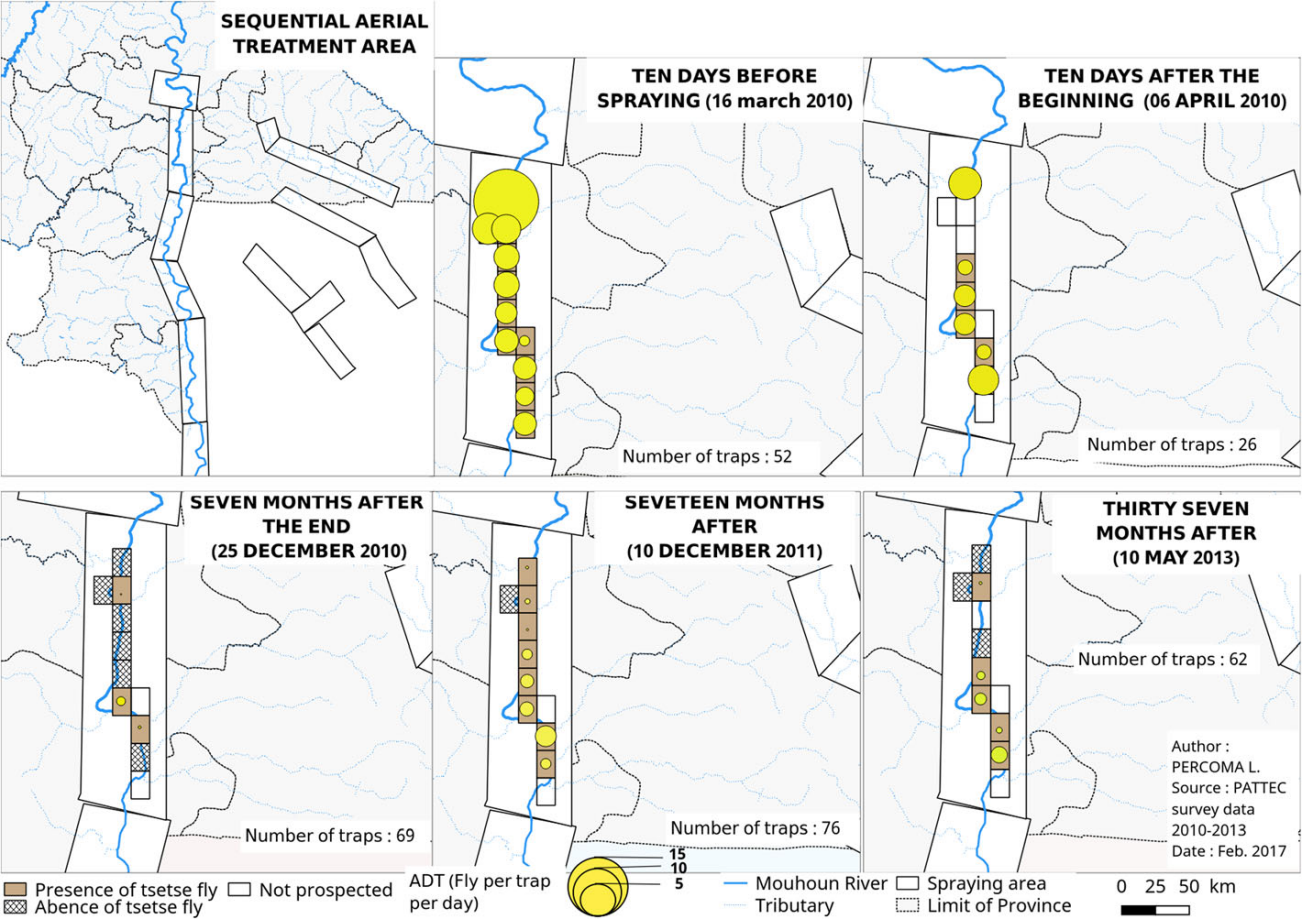
Dynamics of tsetse apparent densities per trap per day (ADT) before and after aerial spraying

Only one species of tsetse fly, *G. tachinoides* was found around the aerial spraying area. Ten days before spraying, the apparent density per trap of tsetse was 4.38 ± 6.01. Ten days after the beginning, 52.63% reduction was noted. At the end of the project, 37 months after the spraying operation, ADT was 0.16 ± 0.56 (96.32% reduction) (Table 5; Fig. 9).

**Table 5:** Impact of aerial spraying on tsetse density

Control	Period	No. of traps	Male	Female	Total	Reduction rate (%)
Before spraying	16/03/10	52	1.68 ± 2.7	2.70 ± 3.6	4.38 ± 6.0	0.00
After 1st cycle	06/04/10	26	0.77 ± 1.2	1.31 ± 1.4	2.08 ± 2.5	52.63
After spraying	25/12/10	69	0.03 ± 0.1	0.01 ± 0.1	0.04 ± 0.2	99.01
	10/12/11	76	0.14 ± 0.3	0.11 ± 0.3	0.25 ± 0.5	94.20
	10/05/13	62	0.09 ± 0.3	0.08 ± 0.3	0.16 ± 0.6	96.32

### Dynamics of trypanosomosis prevalence in sentinel herds

**Fig. 10:**
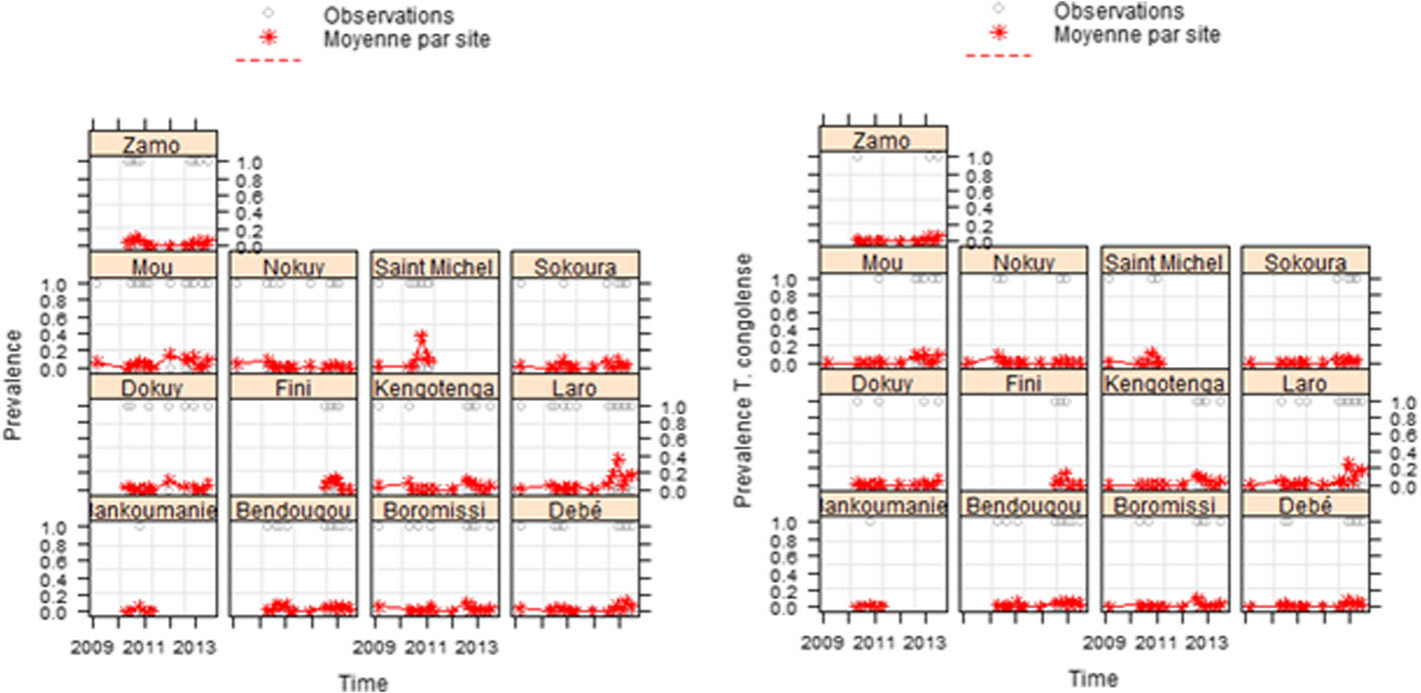
Prevalence of trypanosomosis in the sentinel herds during the survey

A total of 7692 blood samples were taken from 790 animal heads (adults and calves) in 13 villages. A significant increase in the prevalence of mixed infections (*T. vivax + T. congolensis)* was observed over time (Fig. 10; Table 6). Calves were significantly less parasitized than adults (*P* < 0.0001). This increase was mainly observed in the village of Saint Michel (*P* = 0.004). For *T. congolense*, only transmitted by tsetse flies, there was also a significant increase in prevalence over time in sentinel herds. As for hematocrit, a significant decrease was observed during the survey (*P* < 0.0001).

**Table 6:** Fixed-effect coefficients for the AICc-best linear mixed-effects model of the trypanosomosis prevalence

Parameters	Value	SE	*t*-value	*P*-value
(Intercept)	-13.800	2.288	-6.030	< 0.0001
Time	0.001	0.0001	4.344	< 0.0001
Loc Bendougou	0.668	0.635	1.053	0.292
Loc Boromissi	0.409	0.636	0.643	0.520
Loc Debé	0.388	0.638	0.608	0.543
Loc Dokuy	0.043	0.676	0.064	0.949
Loc Fini	0.921	0.662	1.390	0.164
Loc Kengotenga	0.483	0.638	0.757	0.449
Loc Laro	1.370	0.613	2.235	0.025
Loc Mou	1.174	0.616	1.908	0.056
Loc Nokuy	0.183	0.645	0.284	0.776
Loc Saint Michel	1.910	0.619	3.085	0.002
Loc Sokoura	0.289	0.650	0.444	0.657
Loc Zamo	0.508	0.643	0.789	0.430
Status calve	-1.812	0.7622	-2.377	0.017

*Abbreviations*: *Loc* locality, *SE* standard error

## Discussion

The interest of traps and impregnated targets in tsetse control had been shown by Merot et al. [25]. In the PATTEC intervention area, a drastic reduction was obtained only after two months. These results confirm the well-established performance of impregnated insecticide targets reported elsewhere [23], and the effectiveness of beneficiary participation. Indeed, the involvement of beneficiary communities is an important point for the sustainability of the achievements of a control campaign [4, 10]. More than 500 village auxiliaries, 955 department agents of Ministry of Resources of Animals and Halieutic (MRAH) participated in the fight and 75 tsetse controls comities were created in agro pastorals zones. Impregnated target effectiveness in tsetse control had been proved in Ivory Coast [26, 27], Burkina Faso in the pastoral zone of Yalé [2], the zones of Satiri and Padema, the agro-pastoral zone of Samorogouan, and in Hauts Bassins Region [10, 28, 29].

The high densities of targets in some areas were due to the existence of dense patches of *Mimosa pigra* or other abundant vegetation. The use of targets impregnated by the production factory represented an important asset. It not only reduced the work load but also the risk of pollution associated to re-impregnation. The only disadvantage was that they were a single use because of the 100% polyester fabric. The best result of re-impregnation is obtained with fabric composed of 67% polyester and 33% cotton. A re-impregnated test had yielded mixed results denounced by fishermen.

In the present study, the size of the target area and the installation of barriers were innovative and contributed to reduce quickly and drastically the number of flies at a wide scale. A paper is under preparation and will be submitted for publication soon. To measure the impact of the barrier, releases of sterile males were carried out at two distinct points inside and outside the barrier. Recaptures were done with 20 traps inside and 20 outside the barrier. We determined tsetse dispersal and showed that the barrier was efficient.

At the end of the project, tsetse apparent density was higher than the initial one on the Siou River, up to Mali. These results could be explained by insufficient community participation in control and maintenance of targets. Indeed, many targets were removed by communities; those which felled were not redeployed. The reinvasion could originate from untreated sites. A survey done after transversal surveys revealed tsetse presence in four untreated sites at Balavé (2), Padema (1) and Tansila (1). Ground spraying was then applied to reduce tsetse density before impregnated targets deployment.

Although more suitable in savanna areas, aerial spraying was successfully applied in gallery forest. This would be explained by the nature of the gallery not totally closed everywhere, spraying period, which corresponds to the temperature inversion times [30], and tsetse presence on upper parts of the leaves which made then vulnerable to insecticides [31]. However, we did not reach the reduction levels necessary to reach eradication (> 98% reduction of adult females at each cycle) [14].

The use of ground spraying and aerial spraying in ultra-low volume using no-residual insecticide allowed a fast reduction of tsetse fly densities. But a fast return towards the initial densities was observed if other strategies did not take over due to the absence of any residual effect of this method. Ground spraying requires more financial and human resources and may not be effective to treat large areas. But it has the advantage to be selective and can be used by non-experiment staff. It requires several successive treatments to be effective. Aerial spraying depends on weather conditions, which often makes it difficult to respect appropriated schedule. It also requires trained staff and special equipment. It does not promote community participation. Impregnated targets kept infestation at a very low level for 4 years. But this required regular maintenance of targets whatever the season. However, impregnated targets become less effective when tsetse densities become very low [20]. That would explain the absence of significant difference between ADT from the second to the last control period. The study showed the effectiveness of the integrated strategy to reduce tsetse populations but that no method can achieve eradication alone, as was observed recently in Senegal [20] and earlier in Burkina Faso [32].

The use of several strategies or techniques, including the sterile insect technique (SIT), could contribute to achieve the eradication of riverine tsetse species in the intervention area. An example eradication campaign was carried out in Unguja Island, Zanzibar from 1994 to 1996 using Area Wide Integrated Pest Management (AW-IPM) approach with SIT component and tsetse pour-on applications of deltamethrin on livestock for tsetse population suppression. Before 1994, trypanosomosis prevalence and tsetse infestation had been reduced by pour-on in livestock abundant area and impregnated target at a density of densities of 40–70 per km^2^ in areas where livestock was absent [33].

An increase of TAA prevalence was observed despite tsetse control. This may be explained by the integration of new animals in the sample, with animal movement from other tsetse infested sites. Indeed, 335 new animals were integrated to the survey during its course. Among them, 4.48% (*n* = 15) were infected by TC, 1.75% (*n* = 6) by TV and 0.30% (*n* = 1) by TC and TV. This result might also be due to transmission by mechanical vectors, which are abundant in this area [34]. Indeed, it has been demonstrated that tabanides can transmit both *T. vivax* and *T. congolense* in this particular area [35, 36].

The Bobo mass-rearing insectary under construction is therefore a considerable asset for the country to achieve this eradication in the future. Recent experimental field release of gamma sterilized male *G. p. gambiensis* showed that their competitiveness might allow their use for an area-wide integrated pest management campaign with a sterile insect component in Burkina Faso [37].

To ascertain sustainability, PATTEC should sustain the efforts and reinforce the commitment of these beneficiary communities by creating and equipping village tsetse control committees with impregnated targets. The organization of regular tsetse fly monitoring and the transfer of some control activities to perennial structures, such as provincials or regionals directions of livestock, would also be beneficial. That justified the creation of “Insectarium de Bobo-Dioulasso - Campagne d’Eradication des Tsé-tsé et Trypanosomoses (IBD-CETT)”.

## Conclusions

Impregnated targets such as aerial or ground spraying have resulted in a drastic reduction of tsetse flies, but without achieving elimination. None of these methods can therefore individually achieve eradication. They need to be integrated with other methods. This release could be started from 3 months after the setting of impregnated targets or immediately after spraying. The significant reduction achieved will result in improved animal productivity. Now in Burkina Faso, PATTEC need to maintain the present suppression status until the development of a tsetse colony big enough to allow the use of the sterile insect technique for eliminating the residual tsetse populations in the intervention area, as it was done elsewhere in Africa [38, 39]. It is the main challenge for the sustainability of the results. To do so, the continuous maintenance of the barriers, the permanent participation of the beneficiary communities and the continuous support from the national decision makers will be essential, as well as a sequential progression of the control activities towards the south, within a regional perspective.

## Additional files

Additional file 1: Table S1. Database for Entomological survey of aerial spraying. (CSV 20 kb)

Additional file 2: Table S2. Database for the periodical control. (CSV 244 kb)

Additional file 3: Table S3. Data for trypanosomosis prevalence. (CSV 362 kb)

Additional file 4: Table S4. Database for the final longitudinal entomological survey. (CSV 62 kb)

## Abbreviations

ADT: apparent density per trap per day
AICc: Akaike information criterion
AU/PATTEC: African Union/Pan African Tsetse and Trypanosomosis Eradication Campaign
AW-IPM: Area Wide Integrated Pest Management
*Gpg*: *Glossina palpalis gambiensis*
*Gt*: *Glossina tachinoides*
IBD-CETT: Insectarium de Bobo-Dioulasso - Campagne d’Eradication des Tsé-tsé et Trypanosomoses
LAB: Left ascendant branch
LDB: Left descendant branch
MRAH: Ministry of Resources of Animals and Halieutic
PATTEC: Pan African Tsetse and Trypanosomiasis Eradication Campaign
RAB: Right ascendant branch
RDB: Right descendant branch
SD: Standard deviation
ULV: Ultra-low volume
UTM: Universal Transverse Mercator coordinate system
